# Espresso Coffee for the Treatment of Somnolence in Parkinson’s Disease: Results of *n*-of-1 Trials

**DOI:** 10.3389/fneur.2016.00027

**Published:** 2016-03-08

**Authors:** Joaquim J. Ferreira, Tiago Mestre, Leonor Correia Guedes, Miguel Coelho, Mário M. Rosa, Ana T. Santos, Márcio Barra, Cristina Sampaio, Olivier Rascol

**Affiliations:** ^1^Clinical Pharmacology Unit, Faculty of Medicine, Instituto de Medicina Molecular, University of Lisbon, Lisbon, Portugal; ^2^Laboratory of Clinical Pharmacology and Therapeutics, Faculty of Medicine, University of Lisbon, Lisbon, Portugal; ^3^Departments of Clinical Pharmacology and Neurosciences, University Hospital of Toulouse and Clinical Investigation Center INSERM CIC9302 and UMR825, University of Toulouse III, Toulouse, France

**Keywords:** Parkinson’s disease, daytime sleepiness, caffeine, *n*-of-1 trials, sleep

## Abstract

There is limited information available concerning the treatment of daytime somnolence associated with Parkinson’s disease (PD); the most frequently applied therapeutic strategies include decreasing the dose of dopamine agonists or adding potential wake-promoting agents. There is recent data from a placebo-controlled trial concluding on a non-significant trend in favor of caffeine. We aimed to evaluate the efficacy of espresso-coffee in the treatment of daytime somnolence in PD. To evaluate the efficacy of espresso-coffee in the treatment of daytime somnolence in PD, we have conducted multiple single-patient (*n*-of-1) clinical trials comparing regular espresso coffee to decaffeinated coffee in PD patients presenting moderate to severe daytime somnolence defined as an Epworth Sleepiness Scale score >9. Each single-patient (*n*-of-1) trial included a sequence of three crossovers (two treatment periods separated by two days of washout). Four patients were included in the studies and three completed the three pairs of treatment periods. In two of the four patients, espresso coffee was considered beneficial. This study concludes that multiple single patient trials are feasible in PD and suggests that espresso-coffee may have a beneficial effect on daytime somnolence in some patients. These results cannot be generalized beyond the patients included in these trials.

## Introduction

Daytime somnolence is a frequent problem in Parkinson’s disease (PD). (1). Clinical and epidemiological data suggest that many factors may disturb sleep quality and/or induce sedation in PD patients. Some may be related to the disease itself (nighttime motor, psychiatric handicap, or pain) or to comorbidity (depression) or aging. Moreover, these may also be linked to direct or indirect effects of the drugs prescribed to these patients, such as antiparkinsonian dopaminergic drugs, and non-antiparkinsonian comedications that affect sleep mechanisms (antidepressants, hypnotics, antipsychotics, antihypertensives, etc.). The respective role of all these contributing factors may also vary from one patient to another, and it is often difficult to assess and conclude whether the effects of a given drug on sleep or alertness results from a direct or an indirect mechanism involving one or several of these factors. Although it represents a relevant problem for the management of PD patients, no treatment intervention other than the control of the precipitating factors has shown a clear benefit. Recently, an European Federation of Neurological Societies/Movement Disorder Society – European Section (EFNS/MDS-ES) therapeutic review suggested Good Practice Points advice to reduce daytime somnolence either by decreasing the dose of dopaminergic drugs, switching to other dopamine agonists, or by adding wake-promoting agents like methylphenidate (2, 3). Despite this, the only Level B recommendation is to add Modafinil, which is the only pharmacological intervention that has been specifically studied for the treatment of excessive daytime somnolence (EDS) in PD patients (4–9). A recent placebo-controlled trial reported a non-significant trend for caffeine, up to 200 mg BID, to improve excessive daytime sleepiness in patients with PD (10). Furthermore, no other potential psychostimulant drugs (selegiline, amphetamines, methylphenidate) or any other non-pharmacological treatments have yet been properly evaluated for their ability to treat daytime somnolence in PD patients.

Therefore, to approach this problem, we designed multiple single patient trials to find out if espresso-coffee has any benefit in daytime sleepiness in PD patients.

## Materials and Methods

### Study Design and Patients

This was a series of multiple crossover clinical trials performed in one site (Lisbon, Portugal) with a run-in period of 1–2 weeks and a planned duration of 10 weeks. The study included four single-patient (*n*-of-1) trials, which were randomized, placebo-controlled, double-blind, and multi-crossover, which were designed to evaluate the efficacy, tolerability, and safety of regular espresso coffee compared to decaffeinated coffee in PD subjects with EDS. A secondary objective was to assess the usefulness and applicability of clinical trials with a single-patient design to evaluate the efficacy of therapeutic interventions in PD.

Parkinson’s disease patients presenting moderate to severe daytime somnolence were selected from the movement disorders outpatient clinic of the Lisbon University Hospital. Patients were considered eligible if they fulfilled the following criteria: diagnosis of idiopathic PD according to the UK Brain Bank (11), aged 30 years or above, have a modified Hoehn and Yahr stage <5 in the “OFF” state, been on a stable dose of all antiparkinsonian drug treatments for at least 1 month, and have a daytime somnolence defined as an Epworth Sleepiness Score higher than 9 (4). Non-eligibility criteria were: intake of antidepressants or anxiolytics that had not been provided at a stable dose for at least 1 month (dose had to remain stable during the study); relevant medical diseases, malignancy or other progressive neurological disorder; clinically significant or unstable arterial hypertension or ECG abnormalities; cognitive impairment as defined by mini-mental state examination (MMSE) score ≤24; clinically significant psychiatric illness, including previous hallucinations or psychotic symptoms; history of alcohol or coffee abuse (more than six cups daily) or other substance abuse within the past 2 years; patients with migraine or other headache types related to the consumption of coffee; and patients who were unlikely to comply with a coffee intake suspension.

The study was approved by the Ethics Committee Board of The Faculty of Medicine, University of Lisbon, and all participants gave written informed consent.

### Procedures

**Figure 1:**
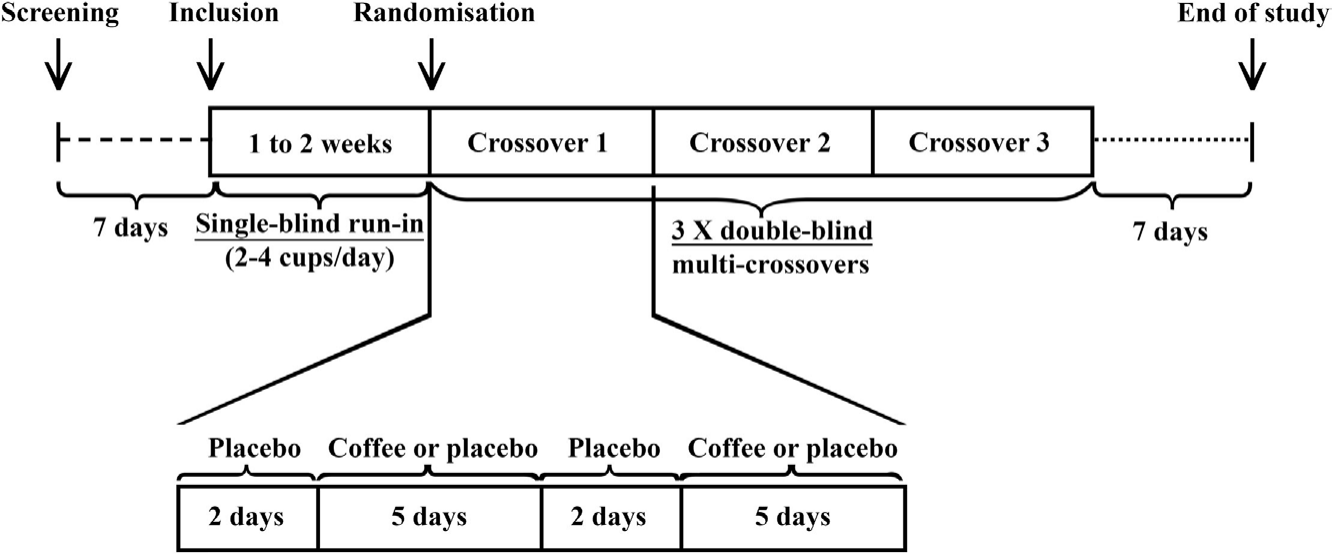
**Study design**.

Each crossover lasted 2 weeks and was composed of two treatment periods preceded and separated by 2 days of washout (Figure 1). Each treatment period corresponded to 5 days of treatment A or B (A – regular coffee, B – decaffeinated coffee). The order of the two treatment periods within each pair was randomized. Following a screening visit to ensure that subjects met all enrollment criteria, subjects started a regular coffee run-in period of 1 week. During this run-in period, subjects took two to four regular espresso coffees daily. Eligible subjects, who maintained a stable consumption of coffee without relevant adverse effects during the run-in period for at least 4 days, were randomized to a multi-crossover sequence. Follow-up visits were planned with 7-day intervals. During the preliminary single-blinded run-in period, the patient was not aware whether he or she was receiving coffee with or without caffeine. If intolerable side effects occurred, the dose of caffeine was titrated to a minimum of two cups a day. If the adverse effects persisted with this lower dose, the patient was dropped from the study.

Each patient received a capsule coffee machine for the purpose of the study. An independent pharmacist was responsible for preparing the randomization schedule by using randomization-generating software.

To reduce the influence of a change of treatment and the possibility that the patient would know the duration and day of onset of each treatment, the patient took the placebo (decaffeinated coffee) during the washout days (Figure 1).

### Outcomes

According to the suggested applications in *n*-of-1 clinical trials (12), the primary efficacy outcome was a 7-point Likert scale monitoring subjective “daytime somnolence”. This questionnaire was completed by the patients on day 5 of each new treatment period and referred to the clinical state during the previous week. Secondary efficacy endpoints were: a 7-point scale monitoring subjective “tendency to sleep” episodes, a 7-point scale monitoring daytime somnolence in the situation/activity in which patients presented the highest tendency to fall asleep, a visual analog scale (VAS) of fatigue, and the Epworth Sleepiness Scale (ESS) (13).

Safety outcomes were: reporting of adverse events, Unified Parkinson’s Disease Rating Scale (UPDRS) Part III (motor) during “ON” state, and UPDRS Part II (Activities of Daily Living, ADL) during “ON” state.

### Investigational Therapy

The therapy under investigation was regular espresso coffee (in espresso machine capsules) as a pragmatic way of delivering caffeine. It was administered as cups of espresso coffee and decaffeinated espresso coffee was given as the “placebo therapy.” The oral cups of espresso coffee were administered two to four times a day. Each cup of regular espresso coffee corresponded to 100 mg caffeine (trademark Platina Coffee, Delta), while a cup of decaffeinated coffee corresponded to 2 mg caffeine (trademark Novadelta Light, Delta). The coffee was packed in unidentified capsules for espresso machines.

### Statistica5l Methods

The first approach to analyze the results was to simply plot the data and examine the results visually, graphically adjusting the randomized sequence of treatments to a rigid sequence of coffee followed by decaffeinated coffee.

For the primary analysis of efficacy on an individual patient, we applied the methodology suggested by Guyatt and coworkers (12). We followed the recommendation that, when analyzing outcomes using a 7-point Likert scale, one should first calculate the difference (“espresso coffee” – “decaffeinated coffee”) for each crossover and afterward calculate the mean difference from the different pairs of treatment periods (*D*). Guyatt et al. proposed that, according to their experience with symptom questionnaires that used a 7-point scale, an improvement of 0.5 points per question corresponded to a noticeable change in the patient’s well-being. As our trial included three questions evaluated with 7-point scales, a total change of 1.5 or higher was considered clinically important. Guyatt et al. also proposed a set of statistical criteria to classify individual *n*-of-1 trials, using a combination of the clinical importance cut-off (0.5 points per question mean difference in symptoms score) and statistical evaluation of the difference observed (one-tailed “*p*” < 0.05). *n*-of-1 trials were classified as beneficial (definitive answer) if “*D*” ≥ 0.5 and “*p*” ≤ 0.05; as tending toward beneficial if 0.5 ≥ “*D*” ≥ 0.3 and “*p*” ≤ 0.05 and CI includes 0.5 or “*D*” ≥ 0.5 and “*p*” > 0.05; as neutral if 0.25 > “*D*” > −0.25, “*p*” > 0.05 and CI < 0.5 or 0.25 > “D” > −0.25, “*p*” > 0.05 and “*D*” for each pair ≤ 0.5 (12).

In addition, a paired student *t*-test was also applied as a global analysis (12, 14, 15). For each crossover sequence, we obtained a single score for each pair by subtracting the mean score of the caffeine period from the mean score of the decaffeinated period. These different scores constituted the data for the paired *t*, and the number of degrees of freedom was set as the number of pairs minus 1. Statistical software programs (SPSS) were used to calculate the *p* value. Each test performed was a one-sided test at a 5% level of significance. The limitations of this test were the assumption of a normal distribution of the data and independence of data from treatment periods. We accepted 80% confidence intervals regarding treatment effects within these *n*-of-1 trials as a compromise between the inherently low power of *n*-of-1 trials having four or fewer treatment pairs and the need to avoid type I (false positive) errors.

Using the same methodology as other researchers, we also defined responders and non-responders according to the number of pairs in which a clear difference between treatments was seen (16, 17). We categorized patients into “responder” (more favorable response to caffeine on the measure by any quantity in all three treatment pairs), “possible responder,” (in two of three treatment pairs), or “non-responder.”

## Results

Four patients were included in the study (Table 1), and three patients completed the three pairs of treatment periods. One patient did not complete the third crossover due to logistical difficulties in performing the last study visits, and only two crossovers were included in the analysis.

**Table 1:** **Patient characteristics at screening visit**.

Patient identification	Gender	Age (years)	Disease duration (years)	Motor fluctuations	Working status	Driving	PD medication (daily)	UPDRS I	UPDRS II ON	UPDRS III ON	HY	SE	ESS
Patient 1	Male	56	5	Yes	Active	Yes	Selegiline 10 mg; Pergolide 2 mg	3	12	30	2	90	19
Patient 2	Male	47	1	No	Active	Yes	Levodopa 150 mg	1	1	13	2.5	100	20
Patient 3	Male	66	10	Yes	Active	Yes	Levodopa 1000 mg	1	18	19	2	70	11
							Selegiline 10 mg						
							Bromocriptine 12.5 mg						
Patient 4	Male	75	6	No	Retired	No	Levodopa 500 mg	6	15	14	1.5	75	13
							Selegiline 10 mg						
							Amantadine 100 mg						
							Ropinirole 6 mg						

*UPDRS: Unified Parkinson’s Disease Rating Scale; HY: Hoehn and Yahr; SE: Schwab and England; ESS: Epworth Sleepiness Scale*.

The recruited patients presented baseline staging Hoehn and Yahr scores between 1.5 and 2.5 and ESS scores ranging from 11 to 20. Other patient demographic and clinical characteristics are described in Table 1.

During the run-in phase, in three patients, treatment was adjusted to three cups of coffee per day, and one patient was up-titrated to four cups daily. All four patients entered the randomization phase.

The visual analysis of the plotted results of the 7-point scales immediately allowed us to distinguish the pattern of response in two patients (patients 1 and 4). They presented a pattern where, within each pair of treatment periods (crossover), they gave higher scores during the regular espresso coffee intake as compared with the alternative treatment in the same crossover. This generates a saw-tooth-like pattern of response, which is suggestive of a beneficial effect. This pattern is visible for the outcomes of global daytime somnolence and somnolence during specific tasks with higher tendency for somnolence, but it was not observed for the outcome evaluating the risk of falling asleep (Table 2).

**Table 2:** **Results of 7-point scale regarding: global daytime somnolence, daytime somnolence in the situation/activity with higher tendency to fall asleep and subjective tendency to sleep episodes**.

		Pair 1		Pair 2		Pair 3		D	Paired *t*-test one tailed	80% CI	Guyatt criteria	No. of pairs favoring coffee	Responder classification
		Coffee	Placebo	Coffee	Placebo	Coffee	Placebo						
Global daytime somnolence	Patient 1	5	3	4	2	5	5	+1.3	0.09	0.08–2.60	Beneficial trend	2	Possibly responder
	Patient 2	6	6	6	7	5	5	−0.3	0.21	−0.96–0.30	No definitive answer	0	Non-responder
	Patient 3	7	6	5	6	–	–	0	0.5	−3.08–3.08	No definitive answer	1	Non-responder
	Patient 4	4	2	4	2	4	4	+1.3	0.09	0.08–2.60	Beneficial trend	2	Possibly responder
Daytime somnolence in the situation/activity with higher tendency to fall asleep	Patient 1	5	4	4	3	4	2	+1.3	0.03	0.70–1.96	Beneficial	3	Responder
	Patient 2	6	6	7	7	7	6	0.3	0.21	−0.30–0.96	No definitive answer	1	Non-responder
	Patient 3	7	7	5	7	–	−	−1	0.25	−4.08–2.08	No definitive answer	0	Non-responder
	Patient 4	4	2	4	2	4	4	+1.3	0.09	0.08–2.59	Beneficial trend	2	Possibly responder
Subjective tendency to sleep episodes	Patient 1	4	4	5	7	7	5	0	+0.5	−2.18–2.18	No definitive answer	1	Non-responder
	Patient 2	7	7	7	7	7	7	0	–	–	No definitive answer	0	Non-responder
	Patient 3	7	7	3	5	–	–	−1	+0.25	−4.08–2.08	No definitive answer	0	Non-responder
	Patient 4	6	2	7	7	6	7	1	+0.29	−1.88–3.88	No definitive answer	1	Non-responder

*Higher scores favor coffee intervention; D: coffee period – placebo period*.

When using the simplest criteria suggested by Guyatt to analyze the benefit of an intervention for a single patient, we concluded that for the primary outcome, the therapeutic intervention was classified as having a “beneficial trend” in two patients. The magnitude of the effect obtained by the combination of the results generated with the three 7-point scales was judged as clinically important in the same two patients (Tables 2 and 3). The same patients were classified as possible responders using other classifications based on the number of paired periods favorable to the active intervention. More favorable and conclusive results were obtained when we analyzed the outcome of daytime somnolence during the activity/tasks that generated higher somnolence (Table 2). In this case, for patient 1, the benefit of espresso coffee reached a statistically significant result with a one-tailed paired *t*-test analysis (*p* = 0.03).

**Table 3:** **Combination of the results of the 7-point scale questions**.

	Daytime somnolence	Somnolence during tasks	Fall asleep	Pooled	Guyatt criteria
Patient 1	+1.3	+1.3	0	2.6	Clinical important result
Patient 2	−0.3	0.3	0	0	
Patient 3	0	−1	−1	−2	
Patient 4	+1.3	+1.3	1	3.6	Clinical important result

When calculating the 80% CI of the estimated difference of the 7-point scale outcome for each single patient, it was favorable toward espresso coffee and excluded zero in patients 1 and 4 for the outcomes of global daytime somnolence and somnolence during a specific task.

When using the ESS scale, no trend was captured (Table 4); however, the evaluation of fatigue using a VAS also suggested a beneficial effect of espresso coffee in patient 1.

**Table 4:** **Results of Epworth Sleepiness Scale and Visual Analogic Scale for fatigue**.

		Pair 1		Pair 2		Pair 3					
		Coffee	Placebo	Coffee	Placebo	Coffee	Placebo	Mean coffee	Mean placebo	Paired *t*-test one tailed	No. pairs favoring coffee
ESS	Patient 1	16	15	14	20	14	16	14.7	17.0	0.18	2
	Patient 2	6	5	6	6	13	15	8.3	8.7	0.37	1
	Patient 3	9	4	9	6	5	Na	9.0	5.0	0.08	0
	Patient 4	13	16	11	7	15	15	13.0	12.7	0.44	1
Fatigue VAS	Patient 1	23	33	23	49	34	49	26.7	43.7	0.03	3
	Patient 2	33	32	7	25	40	38	26.7	31.7	0.26	1
	Patient 3	91	96	6	92	94	Na	48.5	94.0	0.23	2
	Patient 4	71	46	43	29	15	22	43.0	32.3	0.19	1

After study termination, the two patients with positive results felt that taking espresso coffee was beneficial and stayed on the same medication. Interestingly, the patient with only two crossovers to analyze and inconclusive results also thought it beneficial to continue the coffee intake. During the study there was one drop-out not related to an adverse effect, one patient suffered an episode of sleep attack during a period of decaffeinated coffee intake, and one case of each of the following was reported: headache, tremor aggravation, and insomnia during an espresso coffee intake period. In addition, an episode of insomnia between the screening visit and V0, when no study treatment was being taken, was reported. There were neither reports of serious adverse drug reactions nor cases of hypertension or parkinsonism aggravation, and the objective evaluation of the UPDRS did not detect any parkinsonism aggravation.

## Discussion

This study is the first to investigate the efficacy of espresso coffee in the treatment of daytime somnolence in PD, using multiple single-patient (*n*-of 1) clinical trials. We compared the effects of regular espresso coffee to decaffeinated coffee and identified that in two of the four patients studied, espresso coffee was a beneficial therapeutic intervention for EDS. In the other two patients studied, the effect was inconclusive, due in one case it only being possible to complete two crossovers, which limited the power of that study. Insomnia and tremor were identified as potential adverse events that should be of special interest in clinical practice or when planning further studies (18).

This study is the second (19) to report on a set of *n*-of-1 trials conducted in PD patients, reinforcing that it is feasible to conduct this type of trial in the PD population. As anticipated, it was not possible to obtain a mean estimate of effect or a precise estimation of frequency of responders, but data concerning the magnitude of effect to expect, variability of outcomes, and the definition of responder can be learnt from our study. A previous multiple-cross-over *n*-of-1 proof-of-concept study was able to conclude on the potential antiparkinsonian and antidyskinetic effect of naftazone (19). In the field of movement disorders, a *n*-of-1 type of study design was also applied to evaluate the efficacy of two deep brain stimulation targets in a patient with a severe form of Tourette’s syndrome (20).

Current therapy evaluations recommends the use of randomized controlled trials (RCT’s), which are designed to average the effect of treatments across a group of individuals. This was the type of design applied in a recently published trial conducted by RB Postuma and colleagues assessing the effects of caffeine (up to 200 mg BID) upon daytime somnolence (10). In a 6-week randomized placebo-controlled trial, they found a non-significant reduction in ESS score (significant on per-protocol analysis) and an improvement of somnolence on a Clinical Global Impression of Change scale. Although concluding on a trend for an improvement on daytime somnolence favoring caffeine, this study design did not allow the investigators to identify the individual responses or to determine the extent to which an average effect would apply to an individual patient.

Another main question at an early stage of the development of a new therapy is the determination whether there is any beneficial effect of a specific intervention. The efficacy of a novel therapeutic intervention in PD is usually assessed by performing a standard parallel or a crossover trial to evaluate, which demand large studies with great logistical effort and expense. An alternative approach is to verify the existence of responders independently of their frequency, and one of the clinical trial designs that can be used is the single subject research study (21).

A single-patient *n*-of-1 randomized clinical trial involves the analysis of the response of one subject when given two or more treatments over time, based on a randomization scheme (12). PD seems an appropriate disease for the application of this methodology because it is a slowly progressive chronic disease, and if a treatment proves effective, maintenance therapy is likely to be prolonged. This study has shown that *n*-of-1 clinical trials are able to evaluate pharmacological therapeutic interventions in PD and that the incorporation of blinding and randomization is also possible. We have also demonstrated that it is possible to identify responders making this type of study a potential alternative to larger pilot exploratory studies when the objective is to evaluate whether responders exist, independent of the magnitude of effect. Additionally, there is only a minimal need for infrastructural support, namely placebo production and design implementation.

There are, however, weaknesses with this type of trial. *n*-of-1 trials are designed to evaluate individual effects to assess the origins of the variation in type of response instead of estimate the average effect in a population. For this reason, results are analyzed for each patient separately, but the methodology for statistical testing in single-patient trials is limited (22). In our study, for pragmatic reasons, the number of treatment periods was set as three, which constitutes the minimum recommended number of crossovers for this type of trial (12). The 7-point Likert scales, used here, are well placed to capture an effect in multi-crossover studies, as outcomes designed for a specific patient-related problem may be more sensitive to change in these designs. With all the limitations of conducting formal statistical analysis in these studies, the statistical relevance of an effect was shown when applied to a question regarding a specific patient situation (e.g., when the tendency to feel somnolent was higher) and not in questions that were global or non-patient specific. It is worth noting that the multiple crossover design may facilitate the occurrence of a rebound effect due to caffeine withdrawal that could favor the caffeine intervention.

Another limitation of this study was the fact that the decaffeinated capsules were not a truly inert intervention, seeing as each capsule contained approximately 2 mg of caffeine.

Our study did not address also the question of the usefulness of conducting *n*-of-1 studies as compared with the use of these interventions during regular care, and two randomized trials that have done such a comparison have had divergent results (23, 24). A further limitation of this trial was the small number of patients evaluated and the limited number of evaluations per treatment period. As a consequence, a definitive result has not been obtained. Our study has concluded that *n*-of-1 trials are feasible in PD. It also showed that espresso coffee has a beneficial effect on daytime somnolence in some patients. However, this study design does not allow results to be generalized beyond the patients included in these trials.

## Author Contributions

JF contributed to the conception, organization, and execution of the research project, participated in the statistical analysis, and drafted the manuscript, TM, LG, MC, and MR participated in the execution of the research project and reviewed the manuscript, AS contributed to the execution of the research project, executed the statistical analysis, and reviewed the manuscript, MB executed the statistical analysis and reviewed the manuscript, CS and OR contributed to the conception of the research project, designed and critiqued the statistical analysis, and reviewed the manuscript.

## Conflict of Interest Statement

JF has held consultancy functions with GlaxoSmithKline, Novartis, TEVA, Lundbeck, Solvay, Abbott, BIAL, Merck-Serono, Merz, Ipsen; has received grants from GlaxoSmithKline, Grunenthal, Fundação MSD (Portugal) and Teva; has been a member of the European Huntington Disease Network and has been employed by Centro Hospitalar Lisboa Norte, Faculdade de Medicina de Lisboa. TM has been employed by University of Ottawa. LG has been employed by Centro Hospitalar Lisboa Norte. MC has been employed by Centro Hospitalar Lisboa Norte. MR has held member functions with the Ethics Committee of Fundação Champalimaud, and with the Ethics Committee of Centro Académico de Medicina de Lisboa; has held functions as a Scientific Committee Member and Expert of the European Medicines Agency, and has been employed by Laboratory of Clinical and Therapeutical Pharmacology of the Lisbon Faculty of Medicine. AS and MB has been employed by Laboratory of Clinical and Therapeutical Pharmacology of the Lisbon Faculty of Medicine. CS has held consultancy functions with the Committee of Proprietary Medicinal Products and the Scientific Advice Working Party of the European Medicines Agency, TEVA, Alkermes, GlaxoSmithKline, and has been employed by Faculdade de Medicina de Lisboa, University of Lisbon and CHDI Management Inc/ CHDI Foundation. OR has held consultancy functions with Abbott, Addex, BIAL, Boehringer Ingelheim, Impax Pharmaceuticals, Lundbeck, Merck Serono, Movement Disorders Society, Novartis, Oxford Biomedica, Teva, Schering-Plough, UCB and XenoPort; has held advisory board functions with Abbott, Addex, Impax Pharmaceuticals, Lundbeck, Merck Serono, Merz, Novartis, Oxford Biomedica, Schering-Plough, Teva, UCB and XenoPort, with honoraria with Boehringer Ingelheim, GSK, Lundbeck, MDS, Novartis, Teva, UCB and has received grants Agencie Nationale de la Recherche (ANR), CHU de Toulose, France-Parkinson, INSERM-DHOS Recherche Clinique Translationnelle, MJFox Foundation, Programme Hospitalier de Recherche Clinique, Boehringer Ingelheim, Lundbeck, Teva and UCB.

## Abbreviations

EDS, excessive daytime somnolence; EFNS/MDS-ES, European Federation of Neurological Societies/Movement Disorder Society – European Section; ESS, Epworth Sleepiness Scale; HY, Hoehn and Yahr; PD, Parkinson’s disease; MMSE, mini-mental state examination; RCT, randomized clinical trial; UPDRS, unified Parkinson’s Disease Rating Scale; VAS, Visual Analog scale; SE, Schwab and England.
